# Laparoscopic management of isolated metachronous adrenal metastases in a patient with esophageal cancer: a case report

**DOI:** 10.1186/s13256-021-02849-8

**Published:** 2021-07-12

**Authors:** Tarun Jindal, Ankush Sarwal, Pravin Pawar, M. Dhanalakshmi, Neeraj Subedi

**Affiliations:** 1 Department of Uro-oncology, Tata Medical Centre, Kolkata, India; 2 Department of Urology, Nepal Medical College Teaching Hospital, Kathmandu, Nepal

**Keywords:** Adrenal, Esophagus, Laparoscopy, Metastasis

## Abstract

**Background:**

The presence of isolated metachronous adrenal metastasis in patients with esophageal cancer is rare. There is significant controversy regarding the management of such patients. Adrenal metastasectomy has been shown to be of benefit in some reports. Minimally invasive approach, although the gold standard for adrenalectomy, has not been used commonly in a postesophagectomy setting owing to the anticipated technical difficulties. We describe one such case wherein this approach helped in early recovery and long-term survival.

**Case presentation:**

A 59-year-old male of Asian ethnicity presented with an isolated left adrenal nodule, 3 years after an Ivor Lewis esophagectomy for a lower esophageal adenocarcinoma. The biopsy of the nodule was suggestive of metastatic adenocarcinoma. The patient underwent laparoscopic excision of the left adrenal gland.

**Conclusion:**

Adrenal metastasectomy, in postesophagectomy patients can provide good oncological control. Laparoscopic approach, though technically challenging, can provide results equivalent to those of open surgery, albeit with less morbidity.

## Background

Esophageal cancer is an aggressive disease, with almost half of the patients treated with curative intent developing locoregional or distant metastasis within a span of 3 years. In the majority of the cases, the recurrences tend to be multifocal, but rarely only a single organ may be involved [1]. There is significant controversy regarding the management of isolated metachronous metastasis in such patients [2]. There are reports that have shown long-term survival benefits of surgical resection of the involved site [3–5]. In this report, we describe a rare case of an isolated metachronous adrenal metastasis in a patient of esophageal adenocarcinoma that could be resected laparoscopically, resulting in long-term disease-free survival. We also discuss the relevant literature that can help in management of these challenging cases.

## Case presentation

**Fig. 1:**
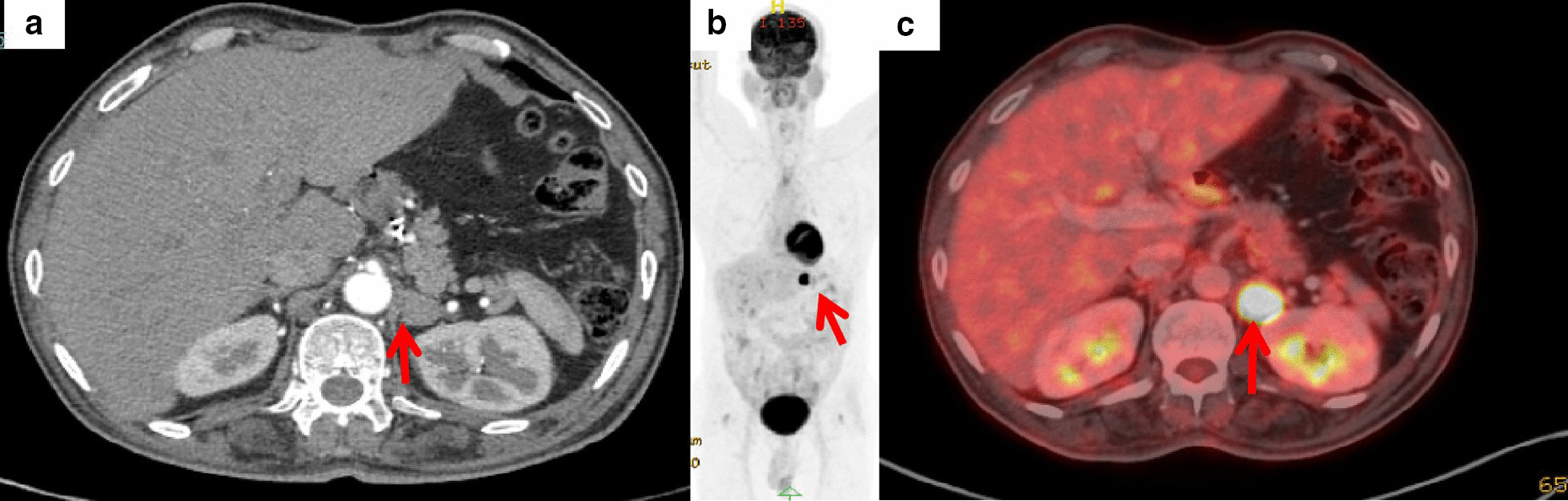
**a** Axial image of the CT scan showing the left adrenal lesion (red arrow); **b** maximum intensity projection image of PET-CT; and **c** axial image showing uptake in the left adrenal lesion (red arrow)

A 59-year-old Asian male who was a shopkeeper by profession presented with complaint of progressive dysphagia to solids that had started a few months ago. His history was suggestive of diabetes and hypertension for the last 10 years. He was on metformin, 1 g twice daily; glimepiride 3 mg once daily; and pioglitazone, 15 mg once daily for diabetes; and telmisartan, 40 mg once daily for hypertension. There was no history of surgical interventions in the past. He was a nonsmoker and nonalcoholic and had no significant history suggestive of acid peptic disease. There was no family history of any malignancies, and his dietary history and environmental history were unremarkable. At the time of initial evaluation, his radial pulse in the right forearm was 88 beats per minute while the blood pressure was 138/90 mmHg. On examination, there was no palpable lymphadenopathy or clubbing. Neurological examination showed normal mental status, no cranial nerve dysfunction, normal reflexes, and normal sensory-motor function. Oral, respiratory, cardiovascular, and abdominal examination was also normal. His blood parameters were as follows: hemoglobin 12.2 g/dl, white blood cell count 10,500/mm^3^, platelet count 277,000/mm^3^, fasting blood sugar 149 mg/dl, urea 18 mg/dl, creatinine 0.47 mg/dl, total bilirubin 0.6 mg/dl, total protein 6.3 g/dl, and albumin 3.2 g/dl. Human immunodeficiency virus (HIV), hepatitis B surface antigen (HBsAg), and anti hepatitis C virus (HCV) antibody tests were nonreactive. Urine routine microscopy was normal while the culture was sterile. An upper gastrointestinal endoscopy revealed an ulceroproliferative growth in the lower esophagus. A biopsy was performed that was suggestive of adenocarcinoma. Positron emission tomography–computed tomography (PET-CT) showed a metabolically active mass in the distal esophagus without any evidence of distant metastasis. The patient was started on preoperative chemotherapy as per the Medical Research Council Adjuvant Gastric Infusional Chemotherapy (MAGIC) protocol. The chemotherapy protocol was as follows: on day 1, he was administered one capsule of aprepitant 125 mg; dexamethasone injection, 16 mg in 100 ml of normal saline administered over 15 minutes; palonosetron injection 0.25 mg intravenously; epirubicin injection, 90 mg intravenously over 5 minutes; oxaliplatin injection, 250 mg intravenously in 250 ml of 5% dextrose over 2 hours; tablet capecitabine (500 mg), three tablets in the morning and two tablets in the evening, continued for 21 days. On days 2 and 3, tablet aprepitant 80 mg was given along with tablet dexamethasone 8 mg twice daily. His blood sugar levels increased owing to the administration of steroid during the curse of chemotherapy, and hence, human Mixtard insulin had to be added to his medications, 22 units before breakfast and 16 units before dinner. He had an Ivor Lewis esophagectomy in December 2015 after completion of three cycles of neoadjuvant chemotherapy. Histopathology was suggestive of residual adenocarcinoma, ypT2NO. In the perioperative period, he was administered injection of cefoperazone and sulbactam 3 g twice daily, injection of paracetamol 1 g three times daily along with injection of fentanyl through epidural catheter for pain control. The patient made an uneventful recovery. Three cycles of adjuvant chemotherapy were administered, which he completed in March 2016. He was under follow-up when he was found to have a enhancing nodule of 1.6 × 1.3 cm in the left adrenal gland on a contrast-enhanced CT scan in July 2018 (Fig. 1a). A PET-CT scan done in October 2018 showed no other pathological uptake except in the left adrenal nodule (Fig. 1b, c). A CT guided biopsy was suggestive of metastatic adenocarcinoma. His laboratory reports at this point in time were as follows: hemoglobin 11.1 g/dl, white blood cell count 9100/mm^3^, platelet count 190,000/mm^3^, fasting blood sugar 134 mg/dl, urea 21 mg/dl, creatinine 0.62 mg/dl, total bilirubin 0.71 mg/dl, total protein 6.9 g/dl, and albumin 3.6 g/dl. Serology was nonreactive, and urine examination was normal. The case was discussed in a multidisciplinary meeting, and a left adrenalectomy was planned as it was the only site of metastasis.

**Fig. 2:**
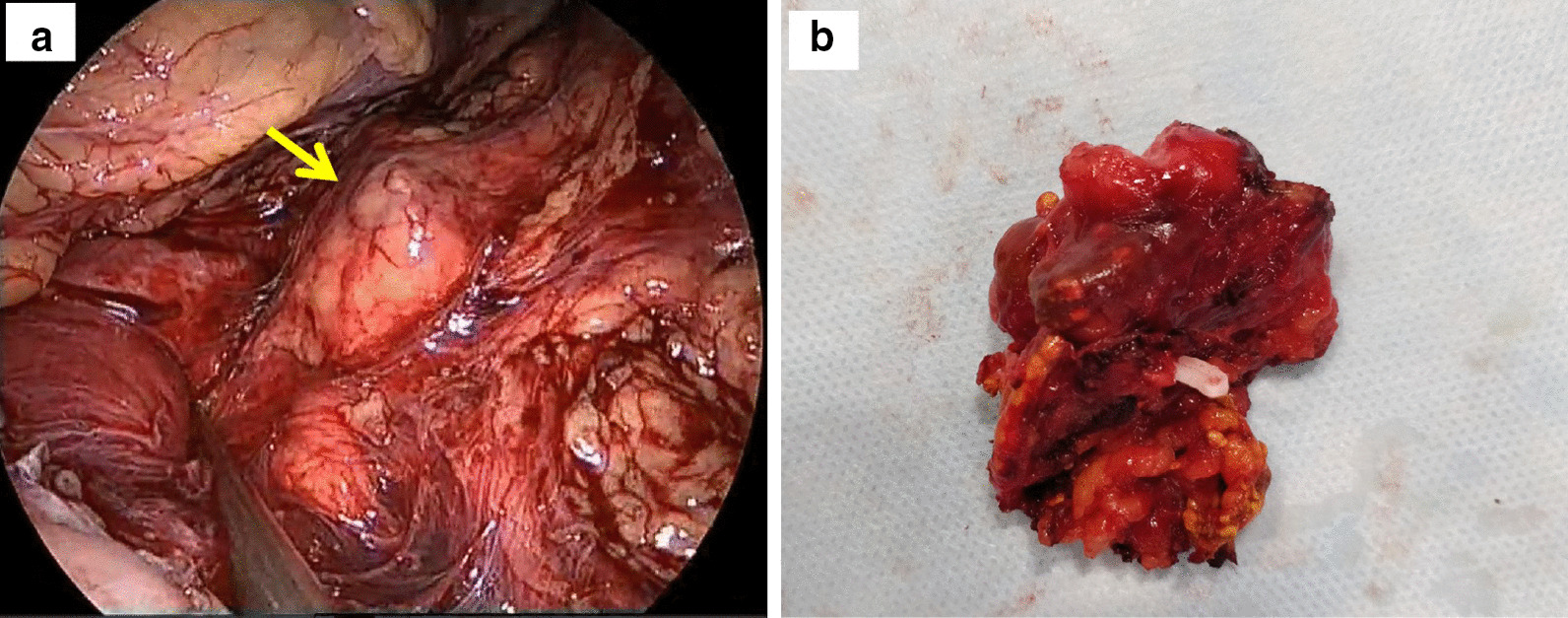
**a** Laparoscopic view showing the left adrenal (arrow) after separating it from the tail of the pancreas; **b** well-encapsulated, excised left adrenal gland

**Fig. 3:**
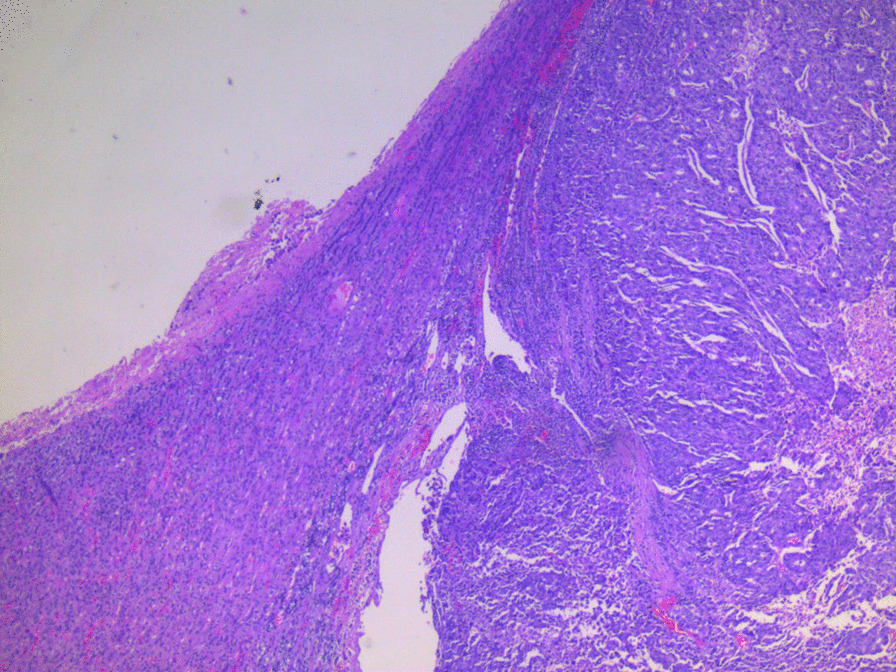
Histopathological image showing the normal adrenal gland on the left side and the focus of the metastatic adenocarcinoma deposit on the right (hematoxylin and eosin, 4× magnification)

The patient was placed in a right lateral position. Pneumoperitoneum was created; the first port was created in the spinoumbilical line, and the abdomen was inspected carefully. There was no visual evidence of peritoneal disease or any other evidence of metastasis; hence, the decision was made to proceed with the adrenalectomy. As there were dense adhesions in the upper abdomen, two more ports were placed in the subcostal and paraumbilical area, taking care to avoid inadvertent visceral injury. The left colon was mobilized, and the retroperitoneal space was entered. The left renal vein was identified, following which the left adrenal vein was isolated and divided. There was significant difficulty in separating the medial aspect of the adrenal gland from the pancreas and the lateral wall of the pulled-up stomach (Fig. 2a). The adrenal gland was then mobilized by blunt and sharp dissection using a vessel sealing device. The gland was bagged and delivered out (Fig.2b). The total operative time was 70 minutes. The patient made an uneventful recovery and could be discharged on the second postoperative day. Histopathological examination of the specimen was suggestive of a metastatic adenocarcinoma focus of 2.9 × 3.3 cm in the background of normal adrenal tissue (Fig. 3). All the margins were negative. On immunohistochemistry, the cells showed uptake of carcinoembryonic antigen (CEA) and caudal-related homeobox transcription factor 2 (CDX2), supporting the pathological diagnosis. The patient was discharged on the second postoperative day. He has successfully completed a follow-up of 27 months and is presently disease-free as per follow-up scans.

## Discussion

We present an extremely rare case of isolated adrenal metastasis from an esophageal adenocarcinoma that presented more than 30 months postesophagectomy. The case highlights the fact that, in such patients, aggressive surgical approach can be beneficial in prolonging the cancer-free survival. Our case also highlights the use of a minimally invasive approach in its management which, though extremely challenging, can offer good oncological and functional outcomes.

Esophageal cancer is an aggressive malignancy. The presence of distant metastasis significantly affects survival. In a review of literature, it was found that 58% of the patients with esophageal carcinoma develop metachronous metastasis and about half of these patients have a single site of metastasis [1]. The usual sites of such metachronous metastases are lungs, nodes, liver, and so on. Isolated metachronous adrenal metastasis from esophageal cancer (postesophagectomy) is extremely uncommon. To the best of our knowledge, only five such cases have been reported in the English literature to date. The majority of these reports have been in patients with squamous cell carcinoma of the esophagus [6].

There is significant dilemma regarding the optimal management of such cases with isolated solid organ metachronous metastasis. A recent systematic review of literature concluded that, owing to the technical advancement in surgical techniques and the acceptable morbidity, the surgical resection of metachronous isolated metastasis to solid organs is a feasible option. It has also been shown to increase the disease-free survival in a selected group of patients [7].

The exact role of adrenalectomy in patients with isolated metachronous adrenal metastasis is unclear owing to its rarity. Almost all of the reports that had patients with squamous cell carcinoma of esophagus with metachronous adrenal metastasis show that adrenalectomy was advantageous [6]. In patients with adenocarcinoma of the esophagus, too, the role of surgery is not established. In a report by O’Sullivan *et al.*, a patient with adenocarcinoma of gastroesophageal junction with postesophagectomy adrenal metastasis had a disease-free survival of more than 4 years following adrenalectomy [4]. Fumagalli *et al.* describe a case of adenocarcinoma of gastroesophageal junction that had a survival of 12 months after excision of metachronous adrenal metastasis [5]. In the present case, too, based on the results of the gastroesophageal junction adenocarcinomas, an adrenalectomy was planned. The patient had good disease-free survival, supporting the notion that adrenalectomy is beneficial in this setting.

Minimally invasive approach is the gold standard for adrenalectomy, but it has been used only sparingly in postesophagectomy settings because of the technical challenges posed by the prior extensive surgery [5]. The left adrenalectomy may prove to be more difficult after esophagectomy and gastric pull-up, as it is closely related to the crus of the diaphragm and the greater curvature of the stomach. A transperitoneal approach is desirable as it offers the advantage of ruling out the presence of peritoneal disease that may not be detected in an imaging study. If difficulty is encountered, a retroperitoneoscopic or open approach can be utilized. It is imperative to follow good oncological principles, and achieving negative surgical margins is of the utmost importance.

## Conclusion

To conclude, surgical resection of an isolated metachronous adrenal metastasis in patients with esophageal cancer can be beneficial. Laparoscopic approach, although challenging, can be safely offered to such patients with good outcomes.

## Data Availability

Not applicable.
